# The Relationship between “Shofuku-Fujin” (Abnormality of Abdominal Examination in Japanese Kampo Medicine) and Body Composition by Bioelectrical Impedance Analysis: A Cross-Sectional Study

**DOI:** 10.1155/2021/6610593

**Published:** 2021-04-01

**Authors:** Tatsuya Ishige, Hiroshi Odaguchi, Toshihiko Hanawa

**Affiliations:** Department of Oriental Medicine Research Center, Kitasato University, 5-9-1 Shirokane, Minato-ku, Tokyo 108-8642, Japan

## Abstract

Shofuku-fujin is an abnormal physical finding in Kampo medical practice. It is assumed to be often found in the elderly and contributes to the selection of Kampo formulas used mainly in elderly patients. However, few objective reports about Shofuku-fujin have been published to date. The aim of this study was to clarify the clinical features of patients showing Shofuku-fujin by using bioelectrical impedance analysis (BIA) and to objectively assess the potential clinical implications of these findings. We conducted a cross-sectional study of 1330 patients who visited our institute to undergo a medical examination by using data collected from September 2010 to March 2016. We extracted data on patient sex and age, anthropometric data, and body composition data that could potentially affect the appearance of Shofuku-fujin. Logistic regression analyses were performed by sex to analyze the various factors related to the appearance of Shofuku-fujin. Of the 1330 patients, the data of 386 men and 942 women were used for analysis. Multivariate logistic regression analysis showed that Shofuku-fujin was associated with older age (odds ratio (OR), 1.07; 95% confidence interval (CI), 1.05–1.10; *p* < 0.001), lower skeletal muscle mass index (SMI) (OR, 0.60; 95% CI, 0.43–0.85; *p* = 0.004), and lower body fat percentage (OR, 0.89; 95% CI, 0.85–0.93; *p* < 0.001) in men and older age (OR, 1.06; 95% CI, 1.04–1.07, *p* < 0.001) and lower body fat percentage (OR, 0.94; 95% CI, 0.92–0.96; *p* < 0.001) in women. On the basis of these results, the factors causing the appearance of Shofuku-fujin were aging, decreased muscle mass, and decreased body fat in men and aging and decreased body fat in women. Our results demonstrated that it may be better to consider a loss of muscle mass when examining a male patient with Shofuku-fujin.

## 1. Introduction

Kampo medicine is a medical system that has been systematically organized based on the reactions of the human body to therapeutic interventions. With its roots in ancient Chinese medicine, this antecedent form of empirical medicine was introduced to Japan in approximately during the 5-6th century. It subsequently developed into a unique form of medicine by adapting to the climate and culture of Japan. A recent investigation reported that about 80%–90% of physicians in Japan had used Kampo medicines in daily practice [1, 2]. In light of the present situation, Kampo medicine is currently taught to students in almost all medical schools in Japan [3].

The most appropriate Kampo formula should be chosen for each individual based on specific diagnostic procedures. The four examinations used for diagnosis consist of inspection, listening, smelling, inquiry, and palpation. Palpation is an examination method in which clinical data are collected from a patient by touching or pressing the body surface. This includes pulse and abdominal examinations. Abdominal examination, Fuku shin, in Kampo medicine is unique to Japan and is a diagnostic procedure for understanding the patient's medical condition and selection of Kampo prescription. Several studies have assessed Fuku shin to date [4, 5].

Shofuku-fujin (Sf) is one of the abnormal findings of Fuku shin in Kampo medicine. It is considered to be the state of jinkyo, which is mainly observed in the elderly. It is a pathological concept that includes the functional decline in the lower body, fluid imbalance, and bone fragility. It includes medical conditions such as dysuria, lower limb weakness, deformities of the lumbar and knee joints, and leg edema. It is also an important finding for the prescription of hachimijiogan (Ba-Wei-Di-Huang-Wan), rokumigan (Liu-Wei-Wan), and goshajinkigan (Niu-Che-Shen-Qi-Wan), which are often administered to elderly patients.

In Japan, which is currently a superaging society, the prevention of conditions that necessitate long-term care and immobilization as a result of frailty and sarcopenia has been recognized as an urgent issue [6]. Sarcopenia, which is characterized by loss of muscle mass, low muscle strength, and/or low physical performance, is more common in older adults and has been considered to be a precursor or the physical manifestation of frailty [7]. Its presentation is frequently similar to jinkyo, and the Kampo prescriptions mentioned above have been proposed for the prevention and treatment of this condition [8, 9].

Bioelectrical impedance analysis (BIA) is a noninvasive technique widely used in body composition assessment [10, 11] and evaluations of nutritional [12] and hydration status [13, 14]. BIA is based on the principle that electric current flows at different rates through the body depending on its composition. InBody 720 is a body composition equipment that estimates segmental body composition (arms, trunk, and legs) using different frequencies (1, 5, 50, 250, 500, and 1000 kHz). The estimated values of skeletal muscle mass, fat mass, bone mineral content, and intra and extracellular water volume by BIA have been used in many previous studies. The Asian Working Group for Sarcopenia recommends BIA for evaluation of muscle mass in community-based screening for sarcopenia because of its noninvasiveness, reasonable cost in comparison with other methods, and ease of use [15, 16].

Since Kampo medicine is expected to be applied to sarcopenia or frailty, it is necessary to objectively understand Sf. However, only one study has explored the mechanism underlying Sf [4]. In that report, abdominal echo findings showed that Sf-positive (Sf+) patients had thinner rectus abdominis muscles in the lower abdomen than Sf-negative (Sf−) patients, but the findings did not mention body composition parameters such as muscle mass. Therefore, the purpose of this study was to clarify the body composition characteristics of Sf+ patients by using BIA and providing objective data for this finding.

## 2. Materials and Methods

### 2.1. Study Design

In this analytical cross-sectional study, patients who visited our facility underwent Kampo medical check-up and body composition analysis by BIA. They were then classified into four groups based on sex and the presence of Sf, and the basic statistics of the four groups were compared. Then, we performed multivariate logistic regression analysis with Sf as the explanatory variable and age, sex, and body composition parameters as the objective variables. More details regarding the specific steps are provided.

### 2.2. Patients

This study included 1330 patients who visited the Kitasato Institute Oriental Medicine Research Center for Kampo medical examination from September 2010 to March 2016. The patients were examined by Kampo specialists and underwent body composition analysis with the InBody 720 bioelectrical impedance data acquisition system (Biospace, Tokyo, Japan). The data of pregnant patients were excluded from analysis because these patients were not eligible for assessment with InBody 720; we also excluded patients showing missing values in examination or measurement data.

### 2.3. Shofuku-Fujin

**Figure 1:**
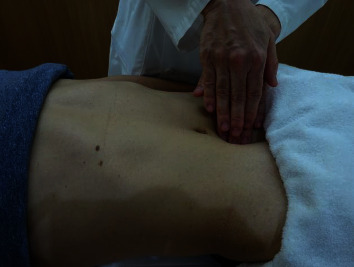
Detection of Shofuku-fujin. Patients were examined in the supine position with their legs extended. Shofuku-fujin is diagnosed when the doctor palpates the patient's lower abdomen with the hands, and the tension on the abdominal wall is clearly weak and easily collapses. Empirically, it is assumed to appear with aging or chronic debilitating conditions.

Figure 1 depicts the detection of Sf. It is generally detected as a loss of tone below the umbilicus and/or hypoparesis in the lower abdominal region in Kampo medicine [17, 18]. In this study, only loss of tone was used to assess this finding, and we did not evaluate hypoparesis because this finding is not routinely inspected by Kampo therapists and is difficult to evaluate objectively.

### 2.4. Measures of Body Composition

The InBody 720 system was used to assess body composition [19]. The study participants stood on two metallic electrodes and held metallic grip electrodes. InBody 720 automatically estimates weight, body mass index (BMI), body fat percentage (BFP), bone mineral content (BMC), intercellular water volume, extracellular water volume, and lean mass of the arms and legs. On the basis of the segmental body composition and muscle mass, the skeletal muscle mass index (SMI) and the ratio of extracellular water divided by total body water (ECW/TBW) were determined and used for further analysis. SMI (kg/m^2^) was calculated by dividing the limb skeletal muscle mass (kg) by the square of the height (m^2^).

### 2.5. Statistical Methods

Statistical analysis was performed using *R* software (version 4.0.2 for Windows 64 bit). All analyses were performed on the basis of sex because previous reports have shown sex-related differences in body composition parameters, including SMI and fat mass index [10, 20]. For all analyses, a *p* value < 0.05 was considered statistically significant.

### 2.6. Clinical Characteristics and Body Composition Parameters

Age, anthropometric measurements, and body composition parameters were expressed as the median and interquartile ranges. Baseline characteristics between the Sf-positive (Sf+) and Sf-negative (Sf−) groups were compared using the Wilcoxon rank-sum test. Then, we calculated the average rates of Sf+ in each decile group of the measured values, displayed them in a bar chart, and examined their relationships using the logistic regression analysis described in the next section. In addition, we analyzed the sex-related differences in the rate of Sf+ in the elderly aged over 65 years by a chi-square test. As a statistical assumption, we confirmed that there was no significant difference in age distribution between sexes by Welch's *t* test (*p* = 0.40).

### 2.7. Logistic Regression Analysis

We first conducted univariate logistic regression analyses and then performed multivariate logistic regression analyses to identify the factors that contributed to Sf. In both logistic models, Sf (presence of Sf = 1 and absence of Sf = 0) was used as a dichotomous variable and defined as a dependent variable. In univariate analysis, we selected the following variables as explanatory variables on the basis of clinical plausibility: age, SMI, BFP, ECW/TBW, BMI, and BMC. We predicted that the contribution of ECW/TBW to the logistic regression model would be too small, so we multiplied it by 100 and used it as an explanatory variable. We checked the assumption of linearity by using scatter plots between the log odds of Sf and the average in each decile group of explanatory variables. Univariate analyses were performed using logistic regression models. Thereafter, we conducted multivariate logistic regression analyses to obtain adjusted odds ratios and to control for possible confounding effects among variables. We followed standard methods to estimate the sample size for multivariate logistic regression, with at least ten outcomes needed for each included independent variable. The independent variables with *p* < 0.05 in the univariate logistic regression analysis were selected. The presence of a multicollinearity pattern among these variables was assessed with two methods. First, bivariate correlations were examined between all covariate pairs with a cutoff Spearman rank correlation coefficient of ±0.70. Second, the variance inflation factor (VIF) was calculated for each covariate. We set the cutoff value at 4.0 as the standard for VIF.

## 3. Results

### 3.1. Patient Selection

**Figure 2:**
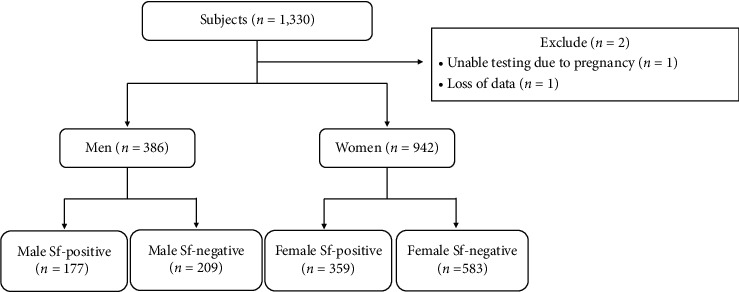
Flowchart of participant inclusion. The patients were divided into four groups according to sex and the presence of Shofuku-fujin. The exclusion criteria included pregnancy and loss of data. Sf, Shofuku-fujin.

Of the 1330 patients, two were excluded, and the data of the remaining 1328 patients were used for analysis. The patients were classified into four groups based on sex and presence of Sf: male Sf+ group, 177 patients; male Sf− group, 209 patients; female Sf+ group, 359 patients; and female Sf− group, 583 patients (Figure 2).

### 3.2. Clinical Characteristics and Body Composition Parameters

**Figure 3:**
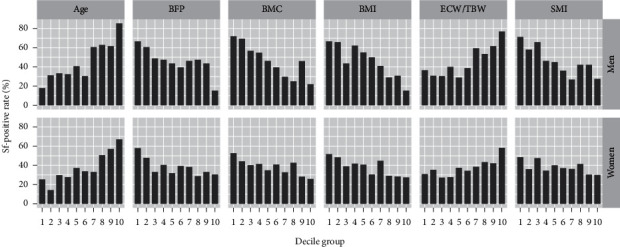
The rate of Shofuku-fujin (%) in each decile group (Q1–Q10) of age and body composition parameters. BFP, body fat percentage; BMC, bone mineral content; BMI, body mass index; ECW/TBW, ratio of extracellular water divided by total body water; SMI, skeletal muscle mass index.

Anthropometric and body composition characteristics of the patients are given in Table 1. Except for ECW/TBW, anthropometric and body composition parameters in the Sf+ group were significantly lower than those in the Sf− group (*p* < 0.05, for both sexes).  Figure 3 shows the average rate of Sf+ by each decile group. In assessments of sex-related differences in the elderly over 65 years, men tended to have a higher rate of findings than women, but no significant difference was observed (74% versus 63%, *p* = 0.13).

### 3.3. Logistic Regression Analysis

**Figure 4:**
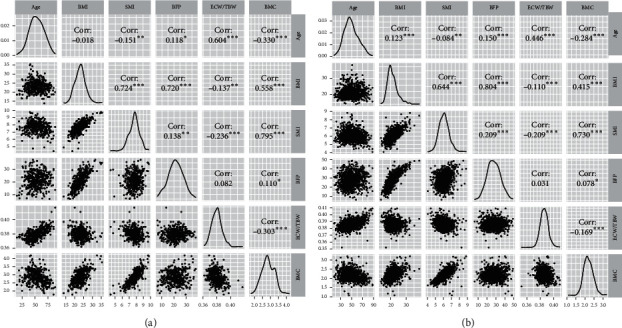
The correlation analysis between explanatory variables by gender. (a) Men. (b) Women. The scatter plot between two variables and correlation analysis by Spearman rank correlation coefficients are shown. ^*∗*^*p* value<0.05; ^*∗∗*^*p* value<0.01; ^*∗∗∗*^*p* value<0.001. Corr, correlation coefficients; BMI, body mass index; SMI, skeletal muscle mass index; BFP, body fat percentage; ECW/TBW, the ratio of extracellular water divided by total body water; BMC, body mineral content.

In the univariate analyses, all six explanatory variables—age, SMI, BFP, ECW/TBW, BMI, and BMC—were significantly associated with Sf in both sexes (all *p* < 0.01) (Table 2). We then performed multivariate logistic regression analysis to adjust for confounding factors. In variable selection, we confirmed linear relationships with a correlation coefficient greater than +0.7 (*p* < 0.05) between each of the five pairs: BMI and SMI (*r* = 0.72, *p* < 0.001), BMI and BFP (*r* = 0.72, *p* < 0.001), SMI and BMC (*r* = 0.79, *p* < 0.001) in men and BMI and BFP (*r* = 0.80, *p* < 0.001) and SMI and BMC (*r* = 0.73, *p* < 0.001) in women (Figure 4). Thus, BMI and BMC were excluded from the models to avoid multicollinearity. In our analysis of the VIF values for the remaining four variables, no VIF values were above 3.0, indicating little, if any, multicollinearity. Therefore, we performed the final multivariate logistic regression analyses with the following variables: age, SMI, BFP, and ECW/TBW. Sf showed significant differences with odds ratios (ORs) of 1.07 ((95% confidence interval (CI), 1.05–1.10), *p* < 0.001) for age, 0.60 (95% CI, 0.43–0.85, *p* = 0.004) for SMI, and 0.89 (95% CI, 0.85–0.93, *p* < 0.001) for BFP in men and 1.06 (95% CI, 1.04–1.07, *p* < 0.001) for age and 0.94 (95% CI, 0.92–0.96, *p* < 0.001) for BFP in women (Table 2). After multivariate adjustment, ECW/TBW did not show a significant association with Sf in men (*p* = 0.52), and SMI and ECW/TBW did not show significant associations in women (*p* = 0.80 and 0.68, respectively).

## 4. Discussion

To our knowledge, this is the first study to objectively investigate the association between Sf and clinical characteristics of body composition in a large sample. We identified the clinical significance of Sf by comparing the results of body composition analysis between Sf+ and Sf− patients. The results showed that male Sf+ patients tended to be older with lower muscle mass and BFP. On the other hand, female Sf+ patients tended to be older and had lower BFP but showed no consistent tendency for muscle mass. Sf did not find a direct relationship with ECW/TBW for both men and women. Among patients aged 65 years and older, men tended to have higher Sf+ rates than women.

A previous study showed that Sf was more often observed in older patients and was associated with less thickness of the rectus abdominis muscle and the linea alba and widening of the space between the right and left rectus abdominis muscles [4]. Sf is thus expected to be associated with aging and a decrease in muscle mass. As expected, we confirmed that Sf was significantly more common with increasing age in both sexes and with less muscle mass in men. However, after adjusting for confounders with the logistic models, Sf unexpectedly showed no clear relationship with muscle mass in women. Several cross-sectional studies on body composition analysis have shown that healthy Japanese women have lower muscle mass and higher fat mass than men [10, 21]. Based on previous reports and the results of this study, we considered that the muscle mass of women was unlikely to affect the presence of Sf because women had a smaller proportion of muscle than men.

Our study also clarified that male Sf+ patients tended to have less muscle mass than male Sf− patients. Muscle loss is often present, especially in elderly people with sarcopenia or frailty. Our results also suggested a strong correlation between skeletal muscle mass and BMC in both sexes (Figure 4). Many reports have shown a strong relationship between skeletal muscle mass and bone mineral density, which was defined as a muscle-bone unit and has been an important concept for simultaneously preventing and treating sarcopenia and osteoporosis [22–24]. To date, research on nutrition and physical activity has actively focused on increasing muscle mass and bone mineral density [25–27]. In this study, we excluded BMC from the final logistic model to avoid multicollinearity; therefore, we could not assess the relationship between BMC and Sf. However, based on previous reports and the high correlation between SMI and BMC, it may be better to provide dietary or exercise guidance to male patients with Sf while paying attention to the decrease in BMC as well as muscle mass.

In addition, we showed that Sf+ patients had a lower percentage of body fat. A pooled analysis of four cohort studies that assessed 4478 Japanese people aged 65 years or older by BIA showed that SMI decreased significantly with age in both sexes and BFP increased significantly with age [11]. Therefore, we considered that subcutaneous fat on the peritoneum was likely to affect the expression of Sf from an anatomical point of view, and obese patients were less likely to have Sf.

In Table 1, the median ECW/TBW was 0.380 for men and 0.386 for women. The normal healthy reference range of ECW/TBW is set to 0.360–0.400 for the InBody BIA device [28]. A comparative study in healthy older persons showed that ECW/TBW increased significantly with age [29]. Some previous reports have shown that ECW/TBW can be an indicator of nutritional status, water balance, or a predictor of locomotive syndrome [29, 30]. In a cross-sectional study of Japanese attending a training gym, the average age and ECW/TBW were 51 years and 0.378 for men and 57 years and 0.385 for women [31]. Since the study was conducted on a population with exercise habits, ECW/TBW was considered to be lower than that of the general population. In a study of 1081 local residents, the average age and ECW/TBW were 64 years and 0.388 for men and 63 years and 0.390 for women [32]. The study showed that about half of women were above 0.390 and that women were more likely to have water imbalance than men, even under healthy conditions. Compared to the previous reports, almost all ECW/TBWs of this study were in normal range, and we considered that they were not necessarily pathological.

ECW/TBW was also expected to be related to Sf when considering the water imbalance in the concept of jinkyo. However, in this study, multivariate logistic regression analyses revealed that ECW/TBW was not directly associated with Sf. Age was considered a confounding factor for them because it met that condition. In fact, ECW/TBW was positively correlated with age (*r* = 0.60, *p* < 0.001) in men and (*r* = 0.44, *p* < 0.001) in women (Figure 4), Sf showed significant differences for age (Figure 3; Table 2), and age could not be an intermediate variable between ECW/TBW and Sf.

From the results of this study, we found that Sf+ patients tended to be older than Sf− patients, resulting in higher ECW/TBW.

We also considered that active treatment with Kampo formulas was probably effective for sarcopenia and frailty. In this regard, animal experiments using Kampo formulas for these conditions have been conducted recently. Two studies have shown that hachimijiogan (Ba-Wei-Di-Huang-Wan) or goshajinkigan (Niu-Che-Shen-Qi-Wan) had a muscle-building effect in mice [33, 34]. Hachimijiogan and goshajinkigan are Kampo formulas that have been used to treat jinkyo in Kampo clinical practice. Since sarcopenia and frailty are pathological conditions similar to jinkyo, these prescriptions are also expected to show improvements in human patients with sarcopenia and frailty. Most Kampo specialists regarded Sf as a necessary finding for their prescribing decisions. On the basis of the findings showing less muscle mass in male Sf+ patients in this study, the prescription of these formulas to yield a muscle-building effect in Sf+ patients appeared to be reasonable. However, their effects on humans have not been clarified. Further research studies, including Sf evaluations, are thus required to investigate the muscle-enhancing effect of these Kampo formulas in human patients.

Several limitations of this study should be acknowledged. First, Sf evaluations would be influenced by measurement bias since it was diagnosed by palpation by medical physicians, and the thresholds for identification of Sf may differ among physicians. Moreover, this was a single-center study, and institution-specific factors may have limited the generalizability of the findings. Second, confounding factors may not be equally distributed between the groups being compared, and this unequal distribution may lead to bias and subsequent misinterpretation.

## 5. Conclusions

In summary, we performed logistic regression analyses using the data of 1328 patients who underwent Kampo medical examination to identify the characteristics of patients by body composition measurement with BIA and to objectively investigate the potential clinical implications of Sf. Our results demonstrated that in comparison with Sf− patients, male Sf+ patients tended to be older and had lower muscle mass and percentage of body fat, while female Sf+ patients tended to be older and had a lower percentage of body fat. In particular, it may be important to pay attention to the loss of muscle mass when examining a male patient with Sf.

## Figures and Tables

**Table 1:** Characteristics of the participants and their body composition parameters.

	Men				Women			
	Total (*n* = 386)	Sf-positive (*n* = 177)	Sf-negative (*n* = 209)	*p* value^a^	Total (*n* = 942)	Sf-positive (*n* = 359)	Sf-negative (*n* = 583)	*p* value^a^
Age (years)	53 (44–63)	60 (50–69)	48 (40–56)	<0.001	48 (40–58)	53 (45–63)	45 (38–53)	<0.001
Height (cm)	170.0 (166.0–173.0)	169.0 (164.6–173.0)	171.0 (167.0–174.0)	0.001	158.0 (154.0–162.0)	158.0 (154.0–161.0)	158.2 (155.0–162.0)	0.025
Bodyweight (kg)	66.3 (60.7–72.1)	62.7 (56.7–68.3)	69.0 (63.5–75.4)	<0.001	51.7 (47.2–57.2)	50.3 (46.0–55.1)	52.9 (48.1–58.3)	<0.001
BMI (kg/m^2^)	23.2 (21.2–24.9)	22.3 (20.1–23.8)	23.9 (21.9–25.8)	<0.001	20.5 (19.0–22.8)	20.1 (18.6–22.1)	21.0 (19.3–23.2)	<0.001
Intercellular water (ℓ)	23.8 (21.8–26.0)	22.8 (20.8–25.0)	24.6 (22.8–26.4)	<0.001	17.1 (15.7–18.3)	16.7 (15.6–18.0)	17.2 (15.9–18.5)	<0.001
Extracellular water (ℓ)	14.6 (13.4–15.9)	14.0 (13.0–15.5)	14.9 (13.8–16.1)	<0.001	10.6 (10.0–11.5)	10.6 (9.9–11.3)	10.7 (10.0–11.6)	0.014
ECW/TBW	0.380 (0.375–0.384)	0.382 (0.377–0.387)	0.378 (0.374–0.382)	<0.001	0.386 (0.382–0.390)	0.387 (0.383–0.391)	0.385 (0.382–0.389)	<0.001
SMI (kg/m^2^)	7.76 (7.28–8.16)	7.54 (7.06–8.02)	7.88 (7.57–8.26)	<0.001	6.06 (5.64–6.47)	6.0 (5.61–6.38)	6.10 (5.70–6.53)	0.008
Body fat percentage (%)	21.3 (17.5–25.5)	20.2 (15.8–24.3)	22.5 (18.7–27.0)	<0.001	27.4 (22.6–32.7)	25.9 (20.8–31.3)	28.0 (23.2–33.2)	<0.001
Bone mineral content (kg)	2.83 (2.58–3.16)	2.71 (2.45–2.96)	2.95 (2.72–3.23)	<0.001	2.17 (2.01–2.36)	2.13 (1.98–2.32)	2.20 (2.04–2.39)	<0.001

The values are presented as median (interquartile range) and grouped according to sex and the presence/absence of Shofuku-fujin (Sf). ^a^Wilcoxon rank-sum test between Sf-positive and Sf-negative groups. Statistical significance was evaluated at *p* < 0.05. Sf, Shofuku-fujin; BMI, body mass index (bodyweight divided by the square of body height; kg/m^2^); ECW/TBW, the ratio of extracellular water divided by total body water; SMI, skeletal muscle mass index (kg/m^2^), calculated by dividing the limb skeletal muscle mass (kg) by the square of the height (m^2^).

**Table 2:** Univariate and multivariate logistic regression analyses to determine the body composition characteristics of patients with Shofuku-fujin.

Variable^a^	Odds ratio^b^ (95% confidence interval)			
	Unadjusted	*p* value^c^	Adjusted^d^	*p* value^c^
Men				
Age	1.07 (1.05–1.09)	<0.001	1.07 (1.05–1.10)	<0.001
SMI	0.49 (0.37–0.66)	<0.001	0.60 (0.43–0.85)	0.004
Body fat percentage	0.92 (0.89–0.96)	<0.001	0.89 (0.85–0.93)	<0.001
ECW/TBW (odds ratio per 0.01 units)	2.42 (1.77–3.39)	<0.001	1.15 (−0.76 - 1.75)	0.52
BMI	0.79 (0.73–0.86)	<0.001		
Bone mineral content	0.21 (0.12–0.37)	<0.001		

Women				
Age	1.05 (1.04–1.06)	<0.001	1.06 (1.04–1.07)	<0.001
SMI	0.73 (0.59–0.90)	0.004	0.99 (0.76–1.23)	0.80
Body fat percentage	0.96 (0.94–0.98)	<0.001	0.94 (0.92–0.96)	<0.001
ECW/TBW (odds ratio per 0.01 units)	1.74 (1.39–2.19)	<0.001	1.06 (−0.81–1.39)	0.68
BMI	0.91 (0.87–0.95)	<0.001		
Bone mineral content	0.35 (0.21–0.58)	<0.001		

^a^Variables presented are age, SMI, body fat percentage, ECW/TBW × 100, BMI, and bone mineral content. ^b^An odds ratio greater than 1.0 represents increased likelihood in the Shofuku-fujin-positive group versus the Shofuku-fujin-negative group. ^c^Statistical significance was evaluated at *p* < 0.05. ^d^The following variables were adjusted: age, SMI, BFP, and ECW/TBW. SMI, skeletal muscle mass index (kg/m^2^), calculated by dividing the limb skeletal muscle mass (kg) by the square of the height (m^2^); ECW/TBW, extracellular water/total body water ratio; BMI, body mass index (bodyweight divided by the square of body height (kg/m^2^)); Sf, Shofuku-fujin

## Data Availability

The data used to support the findings of this study have not been made available because no informed consent for data sharing was obtained from the participants.
